# The Health Literacy of Hong Kong Chinese Parents with Preschool Children in Seasonal Influenza Prevention: A Multiple Case Study at Household Level

**DOI:** 10.1371/journal.pone.0143844

**Published:** 2015-12-01

**Authors:** Winsome Lam, Angela Dawson, Cathrine Fowler

**Affiliations:** 1 School of Nursing, The Hong Kong Polytechnic University, Hong Kong, China; 2 Faculty of Health, University of Technology, Sydney, Australia; Universidade de Brasilia, BRAZIL

## Abstract

**Background:**

Health literacy influences individual and family health behaviour, health services use, and ultimately health outcomes and health care costs. In Hong Kong, people are at risk of seasonal influenza infection twice a year for three-month periods. Seasonal influenza is significantly associated with an increased number of hospitalized children. There is no research that provides an understanding of parents’ health knowledge and their access to health information concerning seasonal influenza, nor their capacity to effectively manage influenza episodes in household. Such knowledge provides valuable insight into enhancing parents’ health literacy to effectively communicate health messages to their children and support healthy behaviour development through role modelling.

**Methods:**

A multiple case study was employed to gain a multifaceted understanding of parents’ health literacy regarding seasonal influenza prevention. Purposive intensity sampling was adopted to recruit twenty Hong Kong Chinese parents with a healthy three-to-five year old preschool child from three kindergartens. A content analysis was employed to categorize, tabulate and combine data to address the propositions of the study. Comprehensive comparisons were made across cases to reveal the commonalities and differences.

**Results:**

Four major themes were identified: inadequate parents' knowledge and reported skills and practices related to seasonal influenza prevention; parental knowledge seeking and exchange practices through social connection; parents’ approaches to health information and limited enabling environments including shortage of health resources and uneven resource allocation for health promotion.

**Conclusions:**

The findings recommend that community health professionals can play a critical role in increasing parents’ functional, interactive and critical health literacy; important elements when planning and implementing seasonal influenza health promotion.

## Introduction

Health literacy comprises “the cognitive and social skills which determine the motivation and ability of individuals to gain access to, understand and use information in ways which promote and maintain good health” [1]. It enables individuals to exert a degree of control over their decision making which in turn determines health outcomes [2]. Health literacy influences individual and family health behaviour, health services use, and ultimately health outcomes and health care costs [2–4]. Health literacy can be categorized into functional, interactive and critical health literacy [2] that deliver benefits to individuals, families and society. Functional health literacy is supported through reading and writing skills that enable people to function effectively in everyday situations. It involves knowledge of risks, health services and readiness to comply with prescribed actions and participate in population health programs. Interactive health literacy concerns an individual’s capacity to act independently with the motivation and self-confidence to apply new information to solve problems in different circumstances, influence social norms and interact well with social groups. Finally critical health literacy involves the application of advanced cognitive skills to critically analyze information to exert greater control over life events and can lead to building community empowerment to address the social determinants of health.

Research shows that there is an association between low health literacy and poor health outcomes [5–7]. Individuals and communities with low levels of health literacy have been found to have higher rates of health care service use, lower information seeking behavior [8] and lower influenza vaccination up take rates [6] than those with high levels of health literacy. The health literacy of parents has an effect on family life style and behavior [9–11] that directly affects the health outcomes of children in their care [12].

Younger children along with pregnant women, and people with chronic medical conditions are at a higher risk of seasonal influenza infection than other groups in the population [13]. In Hong Kong influenza is significantly associated with increased hospitalization rate of child under five years of age [14]. The numbers of children hospitalized as a result of influenza highlights the need for policy makers to focus on improving existing family health promotion strategies to avert child illness and plan effective preparedness and response systems to address future influenza epidemics [15]

The level of Hong Kong Chinese parents’ health literacy, in terms of parental knowledge and practices in relation to preventing and managing seasonal influenza prevention is unknown. There is no research that provides an understanding of parents’ health knowledge and access to health information concerning influenza, nor their capacity to effectively manage flu episodes in the home. Such knowledge could provide valuable insight into ways of enhancing parents’ health literacy so they can not only effectively communicate health messages to their children but support the development of healthy behavior through role modeling [16]. Parents are well positioned to reinforce their children’s healthy practices through their daily interactions and make informed decisions concerning health care use as they spend significant time with their children. This includes the practice of hand washing and cough etiquette as recommended by the WHO and Hong Kong Department of Health as effective measures to prevent the spread of influenza [17–19]. Apart from these preventive measures, evidence exists supporting the use of facemasks by an influenza infected person to reduce influenza viral transmission [20]. Two studies found that close contact and the longer the time spent with an infected person were strong predictors for contracting an influenza viral infection [21]. While early implementation of correct facemask wearing after the onset of symptoms decreased the chance of viral transmission [22]. This implies that wearing a facemask on its own is not enough to prevent influenza viral transmission. Maintaining an optimal distance of one metre between individuals and prompt facemask wearing are both important to enhance the effectiveness of seasonal influenza prevention [15]. A key strategy in many countries has been the annual provision of an influenza vaccination for high-risk groups such as young children and older people with chronic illness to prevent and reduce influenza transmission and related complications [17, 23–25]. In Hong Kong’s public clinics, free influenza vaccination is only provided to elder people aged over 65 with having chronic illness in public clinics [26]. Children between the age of six months and less than six years may receive subsidized vaccination from private doctors. This might be the reason for only 9% of 401 parents allowing their children to receive influenza vaccination in a local study [24]. Low vaccination rates associated with low parental knowledge of influenza vaccination and perceptions of side effects were also reported in this study.

The Centre of Health Protection of Hong Kong (CHP) [27] has focused on raising public awareness of influenza infections using a variety of methods including printed materials, websites, telephone hotlines, briefing sessions, public television announcements and media interviews, along with large-scale publicity campaigns to promote personal and environmental hygiene, and various vaccination programs [27]. This top-down preventive approach has its limitations and has been challenged for its inability to create lasting behavioural change that reaches all populations [28]. Using this approach, parents are positioned as recipients of information and are not actively involved in family health promotion activities. They are not engaged as stakeholders in identifying health problems, developing health promotion strategies, implementing those strategies and evaluating the implementation process [28]. As a result, they are less likely to feel engaged in health promotion campaigns and unlikely to act upon the messages communicated in these efforts. In a survey finding of 513 Hong Kong citizens by the Public Opinion Program (2013) on hand washing and mask wearing behaviour. Despite considerable health communication campaigns this survey found, around half of the parents did not practice recommended hand washing and mask wearing techniques when they had flu symptoms. Among those parents, one quarter were parents of children aged below 15 years [29].

This paper reports on a study to explore the health literacy of Hong Kong Chinese parents with preschool child/ren regarding seasonal influenza prevention. The aim of this paper is to provide useful insights into the design and delivery of health promotion initiatives to address influenza at both community and population level.

## Methods

### Research Approach

A multiple-case study design was employed to investigate functional, interactive and critical health literacy of parents' seasonal influenza prevention in different family context. A case comprised a parent with preschool child/ren between three-to-five years from a range of preschools and family backgrounds. The approach allowed the researchers to analyze phenomena from multiple perspectives within each real-life setting and across settings to understand the similarities and differences between the cases [30, 31]. A multiple-case study approach is advantageous because it is thought to produce substantial and robust results beyond the limitations of a single case study [30]. This research approach can enable the discovery of previously unknown features regarding seasonal influenza prevention and family health promoting behaviours, providing in-depth knowledge of human realities and ascribed meanings [32–34].

### Participants

Purposive intensity sampling was used to provide rich information that manifested the phenomenon of health literacy among parents with preschool children. A networking approach was used to identify potential preschools. Letters and study information sheets were sent via the person-in-charge of five Hong Kong kindergartens for distribution to Chinese parents, inviting them to participate in the study by contacting the researcher. Finally, three kindergartens agreed to join the study. The researcher then personally invited the parents and provided a detailed explanation of the research. The selection criteria were parents with a healthy three-to-five year old preschool child/ren who were willing to communicate and share their experiences. Twenty mothers and their healthy preschool children from three kindergartens were recruited.

**Fig 1:**
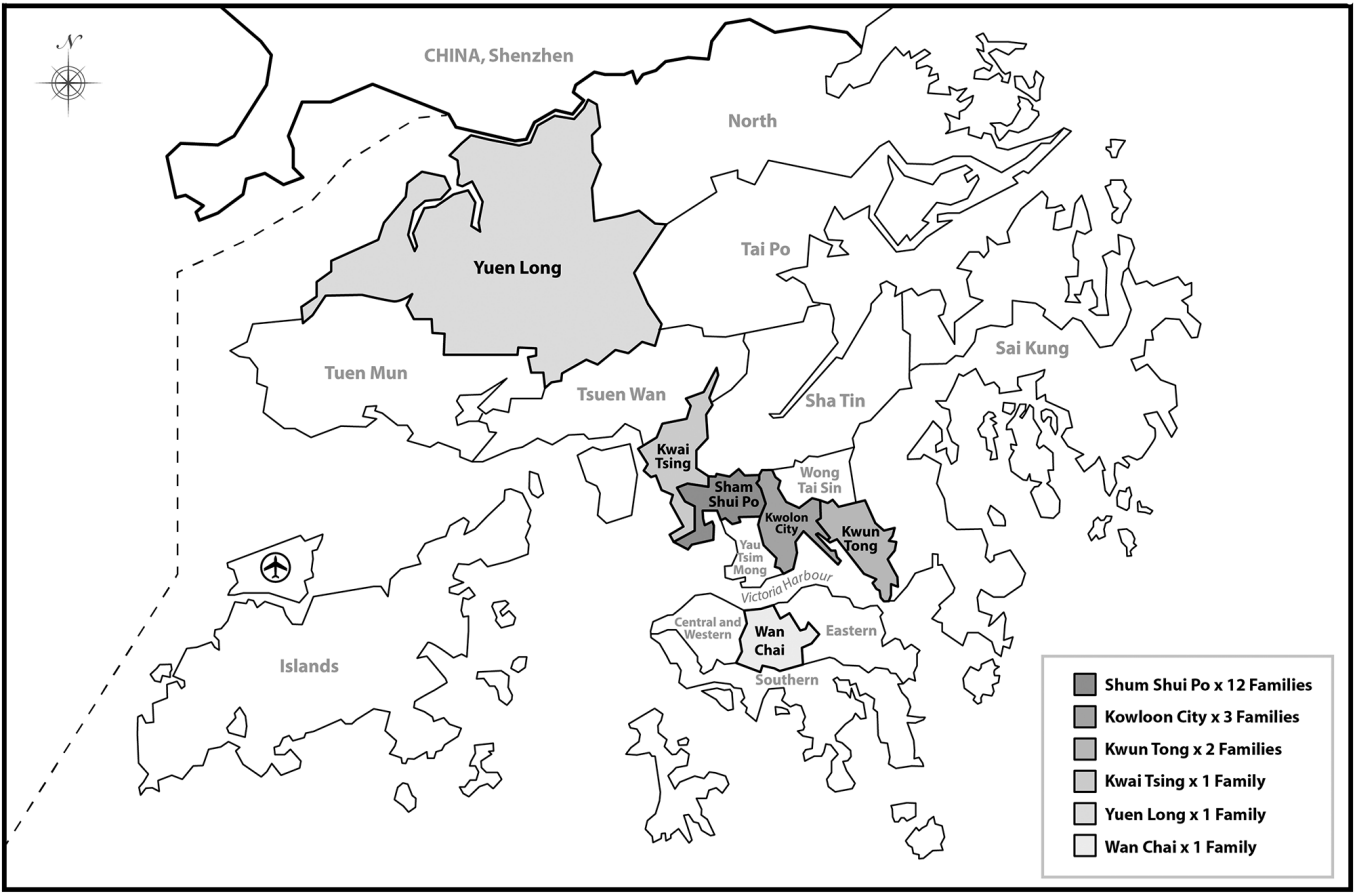
The location of 18 districts in Hong Kong.

The three kindergartens were from three out of 18 Hong Kong districts. They were located in Kowloon City, Shum Shui Po and Wan Chai respectively. Two kindergartens were non-profit and co-educational preschools. One was a private independent and co-educational kindergarten. The 20 families came from six districts. Twelve families were from Shum Shui Po, three families from Kowloon city, two from Kwun Tong and one from Kwai Tsing, Yuen Long and Wan Chai correspondingly (Fig 1).

Twenty participants were mothers and the main caregivers of their children; aged from 28-to-42 years old. Three families had one child; fourteen had two children and three families had three children. Ten mothers had received secondary school education and ten completed tertiary education. Fifteen mothers were housewives and five mothers were full-time workers with their children being cared for by other family members (Table 1).

**Table 1:** Demographic data of 20 families.

Personal particulars of 20 participants:	
Sex of participants	20 mothers
Age of participants	2 were 28 years old
	7 were between 30–35 years old
	8 were between 36–40 years old
	3 were between 41–45 years old
How many children in the families?	3 families had 1 children
	14 families had 2 children
	3 families had 3 children
Employment of main caregivers	15 housewife
	5 full time worker
Who is/are the main caregiver(s) of the child(ren)?	15 children were care by mothers
	1 child was cared by both father and working mother
	4 children were cared by grandmothers and working mothers.
Education of main caregivers	10 mothers received secondary school education
	10 mothers received tertiary education
Monthly household income (HKD)	1 was under 10,000
	10 were 10,000–20,000
	5 were 20,000–40,000
	4 were over 40,000

### Data Collection

Individual semi-structured interviews using open and closed questions were undertaken and field notes were taken to record the observations of parent-child health education activities. The interviews were digitally recorded and then transcribed verbatim for data analysis, with the purpose of understanding parents’ functional, interactive and critical literacy on seasonal influenza prevention and health practices. Open-ended questions to elicit participants’ seasonal influenza health practices and related health promotion activities included: “what strategies/activities do you use at home to increase your confidence regarding promoting healthy behaviours or minimize the risk of developing influenza?”; and “what are the concerns you have (if any) about promoting health behaviours to prevent or minimise seasonal influenza for your child and other family member?” Closed questions sought to collect: demographic data, knowledge of influenza prevention including hand washing, cough etiquette knowledge, mask wearing and related practices. The closed questions were based upon a review of relevant literature that included infection control measures of patients with acute respiratory diseases in community settings [18] and procedures guiding hand washing and facemask wearing [35, 36]. The closed questions were then, validated, using a content validity index, by a panel of academic expert, community nurses and school teachers who were knowledgeable about infection control and child and family health. This index ensured that the questions were technically correct, unambiguous, culturally appropriate and able to provide adequate information to address the proposed research questions [37]. Parents’ knowledge and skills to guide their family’s behaviour in seasonal influenza prevention and promoting healthy behaviour were investigated using a Likert scale with one representing the least adequate to ten indicating the most adequate. The frequency of parents’ hygiene practices were assessed using a five level scale: every time, often, sometimes, seldom and never. Field notes were taken to record the observations during interviews that included noting the tidiness of the home environment, facemask use and the availability of a thermometer, liquid soap and tissues for hand washing and drying, toy storage and parent-child interaction in relation to health practices.

### Data Analysis

A content analysis of the interview transcripts was employed that consisted of examining, categorizing, tabulating and combing the data to address the propositions of the study [30]. First, the researcher reviewed the raw data from the 20 semi-structured interviews and then identified the primary codes by marking and linking them according to the research objectives. The codes were grouped into broad categories through repetitive scanning of the data [30]. Categories within the text that yielded certain themes were collated into theme clusters. Analysis continued until no new themes emerged, as data saturation occurred [33, 38, 39]. A deductive approach was then, applied for pattern-matching [38]. The researcher went back to the original data, read through it repeatedly, compared the derived patterns with previously outlined predefined patterns in order to ensure no missing data and consistency with research objectives. The themes and data extract were independently examined by two other researchers in order to achieve an agreement on patterns matching for the purpose of internal reliability of the study [30]. Exclusions to the thematic codes were also identified and discussed with these researchers.

The demographic, numerical data and observations revealed an overall understanding of parental health practices in seasonal influenza prevention. These data provided a context for parent response enriching the analysis of each case as it provided a context for parent responses.

In the final stage, a synthesis of findings was undertaken [30, 33]. The researcher made a comprehensive comparison across cases including families’ demographic information, living environment, parental health knowledge and practices. This cross-case analysis of the multiple cases provided an understanding of the commonalities and differences between cases in regard to seasonal influenza prevention [30, 33].

### Ethical Considerations

This study was reviewed and approved by the Hong Kong Polytechnic University Human Research and Ethics Committee (reference no.: HSEARS20140121001) and University of Technology Sydney Human Research Ethics Committee (approval number: 2014000072). All participants provided informed written consents were obtained from all parents before the interviews commenced. For the children, written informed consents were also obtained from their parents for the inclusion of children in the observation process. Observations included parent and child face mask wearing, hand washing and cough etiquette practices. Participation in the study was voluntary with an understanding they could withdraw from the interviews at any time.

### Findings

The analysis identified four major themes across the data. These themes were: parents' knowledge and reported skills and practices in relation to seasonal influenza prevention; parental knowledge seeking and exchange practices; parents’ approaches to health information and enabling environments for health promotion.

### Parents' Knowledge and Reported Skills and Practices

In response to the closed questions about the adequacy of parents’ knowledge and skills to guide their family’s behaviour in seasonal influenza prevention and promoting healthy behaviour, thirteen (65%) out of the 20 who responded identified they had adequate knowledge and skills (rated seven-to-eight out of ten points on the scale). Three (15%) scored 6-to-6.5 points as having moderate knowledge and skills. Three participants indicated 5 points and 1 gave themselves 4 points.

However, parent responses to how they perceived their knowledge and skills levels did not concur with their actual health practices descriptions. Considerable gaps were noted in parents’ responses to their perceived hygiene practices with their actual health practices observed during interviews. All parents reported that they washed hands after blowing their nose, coughing and sneezing; before eating and before handling food (Table 2). However, their actual health practices did not concur with their descriptions. (Table 3)

**Table 2:** Knowledge and skills of hygiene practices.

**Knowledge of hygiene practices (N = 20)**	**Frequency of hygiene practices**				
	**No**	**Every time**	**Often**	**Sometimes**	**Seldom**
When do you (parent) wash your hands with soap?					
Before touching the eyes, nose and mouth	9	2	0	8	1
After blowing nose, coughing and sneezing	0	20	0	0	0
Before eating	0	20	0	0	0
Before handling food	0	20	0	0	0
Before touching your child	12	3	0	5	0
After caring for the sick	5	6	0	9	0
After touching public installation such as escalator handrail, elevator control panel or door knobs	11	6	0	3	0
Do you maintain adequate distance (1 metre) between you and the infected person?	13	3	1	3	0
If you do not maintain adequate distance (1 metre) between you and the infected person, do you wear mask?	17	0	0	3	0
Do you clean toys and equipment regularly?	5	4	3	4	4
Do you put on a facemask on while having respiratory symptoms?	3	6	6	4	1
Which of the following is correct thing to do with tissues after sneezing and blowing your nose? Wrap up respiratory secretion with tissue paper and discard it into garbage bins without lids. (N ≠ 20)	0	7	1	0	0
Do you touch your mask once it is secured on your face?	7	1	3	5	4
Do you wash your hands before and after touching the mask?	17	1	0	1	1
Do you change mask at least daily. Replace the mask immediately if it is damaged or soiled?	3	17	0	0	0
Do you know how adequate should the distance be kept between you and the infected person?	Don’t know	18	Know	2	
**Skills on hand washing: (N = 19)**	**Perform**		**Do not perform**		
Backs of fingers to opposing palms with fingers interlocked.	12		7		
Rotational rubbing of left thumb clasped in right palm and vice versa.	4		15		
Rotational rubbing, backwards, and forwards with clasped fingers of right hand in left palm and vice versa	4		15		
Wrists are rubbed	9		10		
Rub all parts of the hands including the wrists with proper hand hygiene technique for at least 20 seconds.	4		15		

**Table 3:** Observations on parent-child interaction in relation to health practices.

Participants	Identified hygiene practices issues	Observations: Parent-child interaction in relation to hygiene practices
P4	Child did not cover mouth and nose when coughing	Son coughed without covering his mouth. Mother gave no response or action to his coughing.
P9	Child did not cover mouth and nose when coughing	Son was playing the digital games. He coughed several times without covering his mouth. Mother gave no response or action to his coughing
P12	Mother did not wash hands before touching her face and nose.	Mother did not wash her hand before and after touching her face and nose for several times during interview.
P14	Mother did not wash hands before taking a pack of lemon tea to her son and did not remind her son to wash his hands before drinking.	Son requested to his mother that he wanted to drink a pack of lemon tea. Mother gave him one pack of lemon tea but did not remind her son to wash his hands before drinking.
P15	Mother did not wash hands before taking a cup of jelly out of the refrigerator for her child and did not remind her daughter to wash hands before eating.	When playing the toys, daughter suddenly requested to eat a cup of jelly. Mother took it out of the refrigerator without washing her hands first. She gave the jelly directly to her daughter without reminding her to wash her hands before eating.
P18	Mother did not wash hands before preparing the bread for her child to eat.	Son told his mother he wanted to eat bread. Mother passed the boy to the domestic helper to help him to wash hand. Mother went to kitchen and took out the bread for her son to eat but she did not wash her hands before touching the bread.
P20	Mother did not remind her child to wash hands before eating.	When playing with the toys, daughter told her mother that she wanted to eat seaweed. Mother asked her to take the seaweed out from the drawer by herself without reminding her to wash her hands. Daughter ate seaweed without washing her hands.

Fourteen (70%) reported they did not or only sometimes washed their hands after caring for the sick. 14 (70%) reported they did not or only sometimes washed their hands after going to the toilet. Eighteen (90%) stated they did not know the distance that should be maintained from an infected person and 14 (70%) reported that they did not put on a facemask every time when experiencing flu symptoms (one parent said they never wore a mask). No parents were able to demonstrate all the steps required for proper hand washing and 15 parents (75%) washed their hands for less than 20 seconds. Thirteen (65%) said that they did not or only sometimes washed their children’s toys. Eight (40%) reported that they discarded used tissues in rubbish bins without a lid (four parents did not know to use a garbage bin with a lid) (Table 2).

In response to the open-ended questions, parents were uncertain when the seasonal influenza peak seasons were and the distance they should maintain from an influenza infected person.

*“I only know it’s around January to March*. *For July and August (summer time)*, *I think that the heat from the sunlight will kill the germs*. *I don’t know it’s also the peak period (of seasonal influenza)*.*” (P20-M83)*

*“I did not pay attention to the distance to be kept from the sick one; I only know that it is advised not to stay too close with them*.*” (P10-M117)*

Parents said they did not wear a facemask throughout the whole disease course.

*“When I start to feel sick*, *I will wear a facemask for the first few days*. *But after taking the medications and my health condition is under control with less sneezing and cough*, *I will stop wearing it*. *I will not wear a facemask throughout the whole disease course*.*” (P17-M74)*

*“I will not wear it if I only have symptoms like runny nose or cough*. *(I will not wear it) throughout the disease course*. *I wear it only in those days my condition is most serious*.*” (P19-M187)*

Parents stated that they would reuse facemasks if they did not look soiled.

*“*[What do you do when you have a facemask on and want to blow your nose?] *I will put the mask on one ear first*, *then blow my nose and wear it back on again*.*” (P17-M94)*

*“I will change the mask once a day*. *Sometimes*, *if my cough is not that serious*, *I will fold the mask up and put it in an envelope for later use*.*” (P19-M212)*

*“If my mask is not too dirty and I do not cough too often*, *I will reuse it (the mask)*. *I will fold it up and use a tissue to wrap around it for later use*.*” (P20- M184)*

The parents identified that they did not always wash their hands properly. Some claimed that washing their hands only with water was adequate for cleaning their hands.

*“The proper procedure of washing hands is very long*. *I only use a very short period of time when washing my hands*. *I will not wash it seriously; the procedure and the time are not enough*.*” (P4-M126)*

*“We usually wash our hands just simply like this*. *We do it in a simple way*. *We do not wash it step-by-step*, *following the instructions*.*” (P14-M306)*

*“If their (children) hands are not too dirty*, *I will not force them to use soap to wash their hands*. *Ha*…*ha…” (P19-M350)*

*“Emm… I basically use it [liquid soap when wash hands] as well*. *But I think that it’s not necessary to use soap all the time because I think it’s already good enough to use water for cleaning hands*.*” (P20-M322)*

Parents reported that they did not know the ratio of water to bleach when performing domestic cleaning. For example:

*“I think it [bleach to water ratio] is really troublesome to follow the ratio of 1*:*99*. *I will just roughly add bleach to a large bucket of water as long as it’s diluted*.*” (P19-M117)*

*“1*:*49 is for cleaning… I don’t remember*. *I cannot differentiate when to use 1*:*99 and when to use 1*:*49*.*” (P20-M88)*

Parents described that they did not have a regular schedule to clean children’s toys. They only cleaned the toys when they were dirty.

*“Sometimes the toys are contaminated by the droplets from sneezing; it’s better to use alcohol to spray them*. *However*, *I did not do so*. *I did not intentionally clean his toys*. *Really!* *” (P10-M150)*

*“I use towel to wipe [their toys]*. *Emm… when they were little*, *I used diluted Dettol to disinfect them*. *But now I seldom clean their toys*. *If their toys have a bit of dust*, *I will use water only to wipe them*. *My husband always says that the best method to clean household is to use water*.*” (P19-M90)*

Parents did not throw their used tissue papers into a lidded rubbish bin. They discarded the tissue in the first available garbage bin. Some parents stated that they had uncovered rubbish bins at homes.

*“When throwing rubbish*, *it depends the location*, *which one is more convenient*, *then I will throw into that bin*. *When throwing*, *I seldom notice whether it [rubbish bin] has a lid or not*.*” (P8-M83)*

*“Because it’s really really dirty*. *If I am at home*, *I will use a tissue to cover my nose when I am sneezing*. *Then I will throw the used tissue into the bin*, *however the bin does not has a lid*.*” (P10-M160)*

### Parental Health Knowledge Seeking and Exchange

Parents reported three common sources for their information seeking and exchange. They were social connection, public service announcement and a community contact point.

#### Social Connection as a Means for Seeking and Exchanging Health Information

The research data illustrated parents’ social connections to different family and community members, social media, the internet and television as sources of health knowledge and practices. Parents identified they accessed knowledge through formal and informal means including direct face-to-face meetings, phone talking, group gathering, attending health clinic and sending messages. The most common method used by parents to share and gain health information communication with other people and parents was through a mobile messaging application. This allowed them to get instant message and exchange messages without having to pay.

*“I know that there is a website for Department of Health*, *but I have not visited it ever before*. *We used to just read information popped up from mobile phone or website*. *We are not used to visiting the website from the government*.*” (P10- M315)*

*“Other parents and I have a WhatsApp group*. *Whoever children are sick*, *the parents will take initiatives to tell one another*. *For example*, *parents will share the health status of their children*, *which disease their children get*? *Then*, *other parents will be posted on the situation and know what to be taken in order to prevent spreading of the disease among the group*.*” (P16-M172)*

For parents a common source of health information was from the internet

*“I will actively look at which type of disinfectant is good*. *I will take a look at books or go to some websites as well*, *those websites for mothers*. *Some topics*, *for example saying that his son is always sick*, *there will be conversations about that for a while*, *and then I will take a look at other people’s experience*.*” (P4-251)*

*“We also share on which type of soup we should boil [to prevent flu or health promotion against flu]*. *I usually go into websites*, *for example Baby Kingdom*. *Inside*, *other users will teach you boiling specific soup to expectorate sputum*.*” (P17-M107)*

In the following quotes, discussion with neighbors and news heard from public media fueled decisions not to vaccinate. These discussions were about complications and side-effects affecting those children who had been vaccinated.

*“I am afraid that there is a negative reaction after vaccination*. *(P6_M101)*

*I am afraid of the reaction after vaccination*. *There are a lot of news which reported the side effects of vaccination*.*” (P6_GM37)*

*“The parent living upstairs said that after flu vaccination*, *her children still got a fever and unwell*. *Therefore*, *I did not let my children receive the vaccination since I am afraid that they will get sick after injection*.*” (P13- M151)*

Parents reported the significance influence of knowledge exchange through intergenerational learning between parent and their elders. The following quotes provide examples of these health promotion messages.

*“When I am looking back to my health practice*, *to certain extend*, *I think my practices are influenced by my parents or family members*.*” (P4-M316)*

*“I heard it* [boiling dried vegetables and pig’s lung soup] *from elder people*… [it] *clear the residue of western medications away from our bodies since they* [medications] *will stay in our bodies after we take them*. *So it’s better to clear them away* [from body]*”*. *(P11-F178)*

*“The other parents share this method*, *eating Bo Ying Dan to prevent influenza with me*. *I also learn it from previous generations*.*” (P20-M194)*

#### Receiving Public Service Announcement

Parents recognized the wider community as a source of health information. School teachers, health professionals and government posters were all identified as health promotion sources:

*“If the school distributes notices back home*, *I will take a look at it… Some kind of notices or anything pamphlets*, *my children take back*, *I will have a look at them*.*”(P3-M203)*

*“There are leaflets in the child and maternal clinic as well as television*. *When we are waiting for doctor consultation*, *the television will broadcast health information on flu*, *sometimes they will teach you about personal hygiene*.*” (P8-M255)*

*“There are posters placed* [regarding prevention of influenza] *at the entrance of library*, *near the places for monthly magazines*. *I did see posters placed there*.*” (P15-P323)*

Parents claimed that their health promotion practices were enhanced through government television advertisements.

*“Every time when I see an advertisement about government providing free vaccinations*, *then I know it is the time to bring them to have vaccinations*.*” (P16-M88)*

*“If it’s the peak season of influenza*, *and when the television broadcasts news about the prevention of influenza*, *I will ask him to wash his hands more frequently*.*” (P11-F40)*

#### A Community Contact Point for Seeking Health Information

Parents described that nursing services could provide a dissemination point for health information to enhance parents’ knowledge and skills in prevention of seasonal influenza.

*“There should be a contact point for nurses to hold health education talks*. *Just like community center*, *at least when the mothers are free*, *they can bring the children to attend the talks*. *If there is no such arrangement in the community*, *we don’t know where to learn and how to prevent seasonal influenza*.*” (P7-M468)*

*“The best method to promote flu prevention is to find a contact point*. *Which means*…*more elder people ask questions in the contact point*, *then they will spread out the learned knowledge/information to others*. *This is the better way*.*” (P11-F254)*

Parents suggested community nursing services might include health consultation. Through the consultation, parents’ misunderstanding and concerns about health issues might be addressed and often reduced.

*“… There are many children living in this estate*. *It is a good if community nurse is here*. *We can get the information from community nurses*. *I remember one of my experiences before; I don’t know what happen to my baby daughter*. *I dialled to the nurse…the maternal and child health centre nearby*. *Phone call is a convenient way to seek advice in emergency occasion*. *It is good to get prompt information*. *At least there is a person who can calm you down first*.*” (P6-M432)*

“[Having a community nurse within the estate] *is convenient for everyone*. *It is convenient for the neighbours; we don’t have to go to clinic which is far away from us*, *for enquiry*. *We can ask her what to eat when having influenza*. *What medication to eat when having a cough*. *Questions like which type of medication is the best will be asked as well*. *Asking the best medications for the fastest recovery of influenza*. *Since we do not know a lot*, *we will definitely ask questions about the type of medications to buy or the best medication first*.*” (P2-M155)*

### Parents’ Approaches to Health Information

The interviews analysis found that parents were interested in learning about health from a variety of sources including: YouTube, government websites, and commercial marketing and were mostly accepting of the information presented. However, the parents had no intention of verifying the obtained health information and potential issues about the reliability and quality of the information were raised:

*“I surf the websites to see anything related*. *Sometimes*, *I will also browse YouTube… I rely on these to obtain information*. *I follow and act on what people say without the need for clarification*.*” (P1-M217)*

*“Those are what the promotion says [they promote health]*. *Ha…Ha… (Laughing)*. *They* [commercials] *will mention about the effect or what will be enhanced physically afterwards*. *Adults like us will take in comparatively less instead but we think that children need more protection; therefore I will buy these* [healthy product] *for them*. *I get no intention to verify the information*, *but sometimes they* [commercial companies] *will quote their sources or from some statistics etc*.*” (P3-M256)*

While some parents trusted the advertisement provided about a product other parents were more critical of the information provided by the commercial company. In the first quote the parent appeared ambivalent about the trustworthiness of a product.

*“I watched the television and they broadcasted their commercial*, *showing that Evergreen H*_*2*_ *O multi purposes disinfectant cleaner is used for mopping the floor*. *I really do not know whether it’s effective or not*. *I really do not think about whether it’s effective or not*.*” (P11-F74)*

In this second quote the parent was much more critical of the impact on her child’s body.

*“Credibility is very important to me*. *My choice definitely is based on its credibility*. *It is very important because it* [Bo Ying Dan] *is taken into their* [children's] *bodies*.*” (P20- M202)*

### Enabling Environments for Health Promotion

#### A Shortage of Health Resources

The parents felt that inadequate health resources provided in the community hindered their health practices. Some parents highlighted the difficulty of finding lidded rubbish bin in the streets.

*“Well…There’s no reason for me to find one with a lid*. *If there is a bin without a lid beside you*, *it doesn’t make sense for you to walk to a further one*. *I know*, *but all the bins near this area do not have a lid*.*” (P2-M78)*

*“If I am on the street*, *I will throw the rubbish to the bin without cover as most of the rubbish bins have no lids*. *I get no choice*. *I only can throw them to these bins [without lids]*.*” (P5_M83)*

Parents complained about the inadequate public hand washing facilities nearby and the lack of liquid soaps in the public toilets.

*“I think the soap provided in the public toilets is not enough*. *There isn’t any all of time*.*” (P15-P304)*

*“We do not have a habit of cleaning his hands after holding the hand rails of escalators*. *I won’t be able to find a washroom to wash hands immediately*.*” (P18-M45)*

Parents emphasised the need to deliver health information resources before disease onset. She recommended broadcasting government health promotion advertisements before the onset of seasonal influenza. It allowed time for parents to improve their preparation to prevent seasonal influenza.

*“Emm… Sometimes I know it* [peak influenza season] *only after watching promotion commercials produced by the government in television*. *Which means I know it only after the flu has come*? *It's too late to prevent*.*” (P14-M270)*

*“Government advertisements start promoting when it has already been the peak period of influenza*. *It has already been too late*. *Government should remind people a bit earlier*.*” (P14-M272)*

#### Resource Allocation for Health

Resource development was identified as disproportionally focused on the health needs of the elderly rather than on children.

*“Yes… The resources of this district are mainly for elderly people*. *It put a large proportion [health resources] on them*. *But I can’t see that they have put any resources on children*. *Apart from vaccination for children*, *I can’t see any resources allocating to them [children]*.*” (P10-F42)*

*“Maybe the resources of Child and Maternal Health Clinic are not enough*. *When my child was up to two years old*, *people there* [in clinic] *told me there is no need to come to clinic again since all follow-up consultations were finished*. *If there is nothing special*, *no need to attend the clinic*. *But*, *I just think*…*I want to know…more on child development or the eating issue of child*…*what I need to pay attention on it*.*” (P16-M261)*

## Discussion

### Enhancing Functional, Interactive and Critical Literacy

Functional, Interactive and critical health literacy can be fostered by increasing parents’ capacities to access and effectively use health information [2] as well as providing health consultation in the community [40]. The internet is a common access point for people searching for health information [41]. Online seeking behavior has become increasingly integrated into the lives of health consumers encouraging them to be active health information consumers [42]. Similar health seeking behaviour was also found in this study. Parents used the internet as a complimentary health resource to search for information [41, 43] and exchanged new health information with other parents. This may have compensated for gaps in the provision of information by health professionals [44], including when parents have difficulty making timely appointments with health professions or when scheduling conflicts arise during clinic hours [45].

Apart from internet-based health education, the use of mobile health technologies such as short message service (SMS) for health message reminder provides new and innovative opportunities for health promotion and disease prevention efforts [46]. This telecommunication for health promotion is feasible in local text as cell phone use in Hong Kong is very common. The SMS application is a cost-effective health promotion method [47] as it will not cost parent’s money when receiving health message. The text messages reminder for seasonal influenza prevention such as childhood vaccination and personal hygiene practices can reach parents in advance. Therefore, parents are able to take preventive measures before seasonal influence comes. In this study, similar health seeking behaviour also reported. Parents claimed that they prefer reading the information popping up from mobile phone.

In order to address parents' needs associated with the change in their seeking behaviours, an electronic integrated health promotion service such as SMS health message reminder and internet-based health education has been recommended as effective ways to facilitate and increase parents' uses of health services [48], and improve parents’ functional literacy [43, 49]. Community nurses could contribute to enhancing parents’ functional, interactive and critical literacy to use health information and service effectively through internet-based health education together with outreach services such as health workshops and consultation session [50]. By participating and learning through workshops, parents’ functional literacy such as health knowledge and skills, and interactive literacy such as developing confidence performing practices independently such as hand washing or coughing/sneezing manner can be further consolidated. Interactive opportunities will challenge parents’ misunderstandings and inaccurate concepts can be corrected. Nurses can play a key role in the provision of educational information, guide parents to websites where reliable health information can be found [51] and provide support as they learn to interpret and integrate online information with traditional health care approaches [43]. These interactive activities enable parents to develop critical literacy skills for the application of health practices in different situations and have greater control of their health through informed decision making [2]. Encouraging parents to participate in the design, development and evaluation of on-line educational programs will ensure educational content is appropriate to the needs of the community [2, 40].

### Process Evaluation and Community Engagement in Program Development

In this study parents raised issues about the absence of adequate enabling conditions that inhibited their ability to take preventative action and to participate in health interventions [52]. These conditions included: the limited availability of hand washing facilities and rubbish bin with lid, access to appropriate child-focused community health services, and community resources for health consultation.

A potential need is for greater community health planning and action that includes: process evaluation before and during planning stages and when implementing seasonal influenza prevention programs [15] in order to address the actual needs of the families. To ensure successful delivery of seasonal influenza prevention within communities, collaboration and partnerships is crucial among community members and public health agencies such as outreach clinics, district councils and schools. This approach ensures adequate health communication and integrates community engagement at every stage of program development [40].

### Strength and Limitation

In this study, the authors did not use Test of Functional Health Literacy in Adults (TOFHLA) or the Rapid Estimate of Adult Literacy in Medicine (REALM) to measure parents’ functional health literacy regarding hand washing and cough etiquette knowledge as they were both developed for English-speaking adult population. The tests are not easily translated to enable assessment of the Hong Kong Chinese populations. The terminologies adopted in the assessment were different from that of other countries because of differences in linguistic systems and sociolinguistic aspects in different countries [53, 54]. The authors took a comprehensive approach by using closed questions and semi-structured interviews to provide a broader perspective of parents’ health practices. The questionnaire developed for this research study may contribute towards establishing a seasonal influenza health literacy assessment for communities.

A limitation of this study is that 20 parents with their preschool children were recruited from six out of eighteen districts. Despite strenuous efforts to recruit a maximum diversity of participants, 12 parents (60%) were recruited from the same district and could be expected to perceive similarly environmental influences on seasonal influenza prevention and related health promotion strategies somewhat, as compared to the parents recruited from other districts.

## Conclusion

A multiple-case study was adopted to examine functional, interactive and critical health literacy of parents' seasonal influenza prevention and related health promotion practices. The findings from this study demonstrated that many parents have insufficient functional and critical health literacy in relation to seasonal influenza prevention and related health promotion. Parents demonstrated a lack of, or incomplete hand washing and mask wearing and a limited critical analysis of available health information obtained from different community organizations. Community nurses could contribute to enhance parental capacity to use health information effectively through internet-based health education together with outreach services. The findings also suggest that community health professions take a critical role in increasing parents’ functional and critical literacy; vital elements when planning and implementing seasonal influenza health promotion intervention.
